# Global trends in ambient particulate matter-attributable ischemic heart disease among the elderly (1990–2021)

**DOI:** 10.3389/fcvm.2025.1653870

**Published:** 2025-10-24

**Authors:** Yangxi Zeng, Lihui Liu, Gang Huang, Can Liu, Xi Lan, Yuhan Mou, Xinyu Wang, Bin Liao, Juyi Wan

**Affiliations:** ^1^ Key Laboratory of Cardiovascular Remodeling and Dysfunction, Department of Cardiovascular Surgery, The Affiliated Hospital of Southwest Medical University, Luzhou, China; ^2^ Clinical Medical College, Southwest Medical University, Luzhou, China

**Keywords:** ambient particulate matter (PM), ischemic heart disease (IHD), global burden of disease (GBD 2021), elderly population, estimated annual percentage change (EAPC)

## Abstract

**Background:**

Airborne particulate matter (PM), particularly PM₂.₅, poses a growing threat to cardiovascular health, especially among older adults. With intensified urbanization and industrial activities, PM₂.₅ has become a major factor contributing to ischemic heart disease (IHD), a leading cause of global mortality. These fine particles can penetrate pulmonary defenses, enter circulation, and trigger inflammatory and oxidative pathways that accelerate vascular damage. The elderly, whose physiological resilience declines with age, are particularly susceptible. While PM exposure's link to IHD is established, patterns by sex and age in older populations remain underexplored. This study assesses global, regional, and national shifts in PM-related IHD burden between 1990 and 2021, identifies disparities across demographics, and examines the influence of socioeconomic development. It also forecasts trends to 2040, providing insights to guide public health interventions for aging societies.

**Methods:**

Using the Global Burden of Disease study (GBD 2021), we analyzed mortality and disability-adjusted life years (DALYs) from 1990 to 2021 across global, regional, and national levels. We calculated age-standardized rates (ASRs) and estimated annual percentage changes (EAPCs). Future trends were projected using a Bayesian age–period–cohort (BAPC) model. Modelling was performed in R, disaggregated by sex and age, linked with PM exposure and socioeconomic development.

**Results:**

Overall IHD due to PM declined globally from 1990 to 2021. The largest reductions occurred in high-SDI regions. Burden increased in low- and middle-income areas in Asia and Africa. Globally, IHD burden from PM was negatively correlated with sociodemographic index (SDI). Men had higher burdens than women, with differences increasing by age. Although BAPC projections indicate a continued decline, a potential rise in pollution around 2035 may slow the rate of decrease or temporarily flatten the downward trajectory.

**Conclusion:**

Although global declines have been observed, the burden of ischemic heart disease (IHD) attributable to ambient particulate matter pollution remains persistently high in Southeast Asia and Africa. Forecasts suggest that elderly individuals, particularly men, will continue to experience disproportionate health impacts in the coming decades. Targeted public health initiatives focused on reducing particulate matter exposure, especially among vulnerable aging populations, are essential to mitigate future cardiovascular risks and promote healthy aging worldwide.

## 1 Introduction

Ischemic heart disease (IHD) is one of the most lethal cardiovascular diseases globally, caused mostly by decreased coronary blood supply, which causes myocardial hypoxia, angina, or myocardial infarction (1). Based on Global Burden of Disease (GBD) 2021 evidence, IHD is still one of the major causes of death worldwide, with its high prevalence and death rates constituting a long-standing challenge to health systems (2). In 2021, approximately 8.7 million deaths, which accounted for 16.2% of all deaths globally, were attributed to ischemic heart disease (IHD) (2). Development and progression of IHD are influenced by an interplay of factors that includes genetic predisposition, unhealthy lifestyle behaviors (such as smoking, poor diet, and physical inactivity), long-standing health status (such as hypertension and diabetes), and environmental factors (3, 4). Over the last few years, there has been a significant amount of evidence that has pinpointed environmental pollution—namely, ambient particulate matter (PM) pollution—as an important causative factor for IHD (5). Fine particulate matter (PM₂.₅), a major component of ambient particulate pollution, is characterized by its extremely small size and complex physicochemical properties. PM_2.5_ particles are small enough to reach the deepest parts of the lung and move into the bloodstream, where they can damage cardiovascular health (6). Ongoing exposure raises the risk of ischemic heart disease (IHD) through multiple biological pathways. It triggers inflammation, with higher levels of interleukins and C-reactive protein (7), and causes oxidative stress that weakens endothelial function and vessel stability (8), speeding up atherosclerosis. It also affects body systems more broadly: PM exposure activates the sympathetic nervous system, increasing heart rate, narrowing blood vessels, and raising blood pressure, while also disturbing glucose metabolism, which adds to cardiovascular risk (9). Together, these mechanisms put lasting strain on the cardiovascular system and increase the likelihood of IHD. These effects weigh more heavily on older adults, who commonly face declining immunity, coexisting chronic diseases, and a gradual reduction in physiological reserve with age (9). As industries expand and cities grow, particulate matter (PM) levels in the air have risen, adding to the burden of ischemic heart disease (IHD) linked with pollution (10). The main sources of PM are not the same everywhere. In low- and middle-income countries, industrial emissions and the burning of solid fuels for cooking or heating are key contributors. In wealthier regions, traffic and power generation are the leading sources. This trend stands out in low- and middle-income countries, where outdoor PM exposure remains elevated and plays a major role in both illness and death linked to IHD (11). As populations age and pollution grows more severe, tracking how the IHD burden from PM changes across places and over time has become vital. Such evidence can help shape timely and effective public health actions.

This work looks at how the burden of ischemic heart disease (IHD) linked to particulate matter pollution has shifted over time, considering differences across regions, ages, and sexes between 1990 and 2021. It also looks ahead, projecting future trends to provide evidence that can guide public health strategies in the coming decades. Through presenting strong evidence regarding the prolonged effects of particulate pollution on IHD, the study intends to guide the creation of more efficient global public health strategies, facilitate targeted measures to reduce pollution-associated cardiovascular risks, and ultimately assist in lessening the worldwide burden of IHD.

## 2 Method

### 2.1 Data source

All empirical data used in this study were obtained from the Global Burden of Disease Study 2021 (GBD 2021). This cross-scalar archive supplies harmonised estimates of mortality and disability-adjusted life years (DALYs) from 1990 to 2021, resolved at global, regional and national horizons. Within the dataset, health metrics are further disaggregated by aetiology, sex and age cohort, and the epidemiological imprint of 87 recognised risk factors is apportioned across these same strata. The modeling framework and validation protocols underlying these estimates have been comprehensively described in previous studies (12, 13). Briefly, we applied a Bayesian age-period-cohort (BAPC) model using integrated nested Laplace approximations (INLA) for parameter estimation. This approach accounts for temporal trends across age, period, and cohort dimensions, and has been validated through out-of-sample prediction and internal consistency checks in prior research.

This study focuses on quantifying the burden of ischemic heart disease (IHD) attributable to ambient particulate matter pollution in the elderly population. We obtained relevant data through the Global Health Data Exchange (GHDx) platform (https://vizhub.healthdata.org/gbd-results/), covering all GBD-defined regions and individuals aged 60 years and older between 1990 and 2021, with ambient particulate matter pollution specified as the exclusive exposure source.

To account for differences in age structure across countries and regions, disease burden indicators were age-standardized using the global reference population distribution adopted by GBD 2021. The method used to calculate age-standardized rates (ASRs) is provided in (Equation 1):

(1) $$ASR=\frac{\sum_{i=1}^{n}a_{i}w_{i}}{\sum_{i=1}^{n}w_{i}}$$

Within the standardisation equation, the subscript i serves as an indexical marker that distinguishes each age cohort, while n registers the total count of those cohorts. For any given cohort, x_i_ corresponds to the pertinent health measure—typically an age-specific rate—whereas w_i_ denotes that cohort's share of the reference population employed in age-standardisation procedures.

To reflect social and demographic conditions, we used the Socio-demographic Index (SDI) created by the Global Burden of Disease (GBD) project. The SDI brings together three measures—average schooling years, fertility rate, and income per person adjusted for inflation—into one summary indicator (14). Countries and regions are then divided into five groups: low, low-middle, middle, high-middle, and high. This approach helps compare regions more directly and reveals differences in health and development.

In the GBD 2021 study, ischemic heart disease (IHD) is defined by both death and disability outcomes, including clinically confirmed and self-reported cases. A Bayesian modeling framework is used to combine data from many sources, spanning countries, age ranges, time periods, and health systems. This makes it possible to estimate disease burden even where local information is limited. Each estimate is reported with a 95% uncertainty interval (UI), based on the 2.5th and 97.5th percentiles of the posterior distribution. Wider UIs point to greater uncertainty, especially in data-poor regions.

The present study explores how ambient particulate matter pollution is associated with ischemic heart disease (IHD) burden in populations aged 60 years and above. Key indicators assessed include mortality, disability-adjusted life years (DALYs), age-standardized mortality rate (ASMR), age-standardized disability rate (ASDR), and estimated annual percentage change (EAPC).These indicators allow for comparisons across time and regions. All data were obtained from the publicly available, de-identified Global Burden of Disease (GBD) database. The GBD protocol received ethical approval from the Institutional Review Board of the University of Washington, and individual informed consent was waived. As this study involves secondary analysis of anonymized data, no additional ethical approval was required.

### 2.2 Statistical analysis

All rate metrics reported in GBD 2021 are expressed per 100,000 population and were age-standardized using the standardization formula described in (Equation 1), based on the GBD standard population structure.

To analyze trends in age-standardized mortality rate (ASMR) and age-standardized disability rate (ASDR) for ischemic heart disease (IHD) attributable to ambient particulate-matter exposure among persons sixty years of age and older, we engaged a series of ordinary-least-squares models over the 1990–2021 archive at global, regional and national scales. Treating calendar year as a temporal axis of inquiry, we regressed the natural logarithm of each age-standardized rate (ASR) against this measure, thereby deriving an Estimated Annual Percentage Change (EAPC) that indexes the pace and direction of epidemiological transformation. The algebraic specifications that scaffold these calculations are reproduced in (Equations 2, 3).

(2) y=α+βx+ε

(3) EAPC=100⋅(exp(β)−1)

In this model, *y* is the natural logarithm of the age-standardized rate [ln(ASR)], *×* is the calendar year, and the slope β represents the average yearly change in ASR. If the estimated annual percentage change (EAPC) and the lower limit of its 95% confidence interval are greater than zero, the trend is interpreted as a significant increase. If the EAPC and the upper limit are both below zero, it indicates a significant decrease. If the confidence interval includes zero, the rate is considered relatively stable.

To better understand these epidemiological changes in relation to social and economic conditions, we analyzed how the burden of ischemic heart disease (IHD) from particulate matter exposure is associated with the Socio-demographic Index (SDI), a marker of regional development. We first calculated Pearson correlation coefficients between SDI scores and burden measures from 1990 to 2021, both worldwide and across the 21 GBD regions. We then modeled linear relationships between SDI and ASMR, ASDR, and their EAPCs for 204 countries and territories, allowing us to observe how disease patterns shift along different levels of socio-economic development.

To project future epidemiological trajectories, we mobilized a Bayesian age–period–cohort framework—implemented through Integrated Nested Laplace Approximations (INLA)—to trace sex-differentiated trajectories of ambient-PM-attributable IHD burden through 2040, an approach repeatedly celebrated for its long-range acuity in chronic-disease forecasting (15). All computational labour took place in R 4. 3. 2: datasets were curated with tidyverse/dplyr, age-standardized projections generated via the BAPC, nordpred and INLA engines, and graphical narratives composed in ggplot2. Statistical inference followed a two-tailed convention, honouring results whose probabilities fell below the 0. 05 threshold.

## 3 Result

### 3.1 Burden of elderly ischemic heart disease linked to ambient PM: global and regional trends (1990–2021)

**Figure 1:**
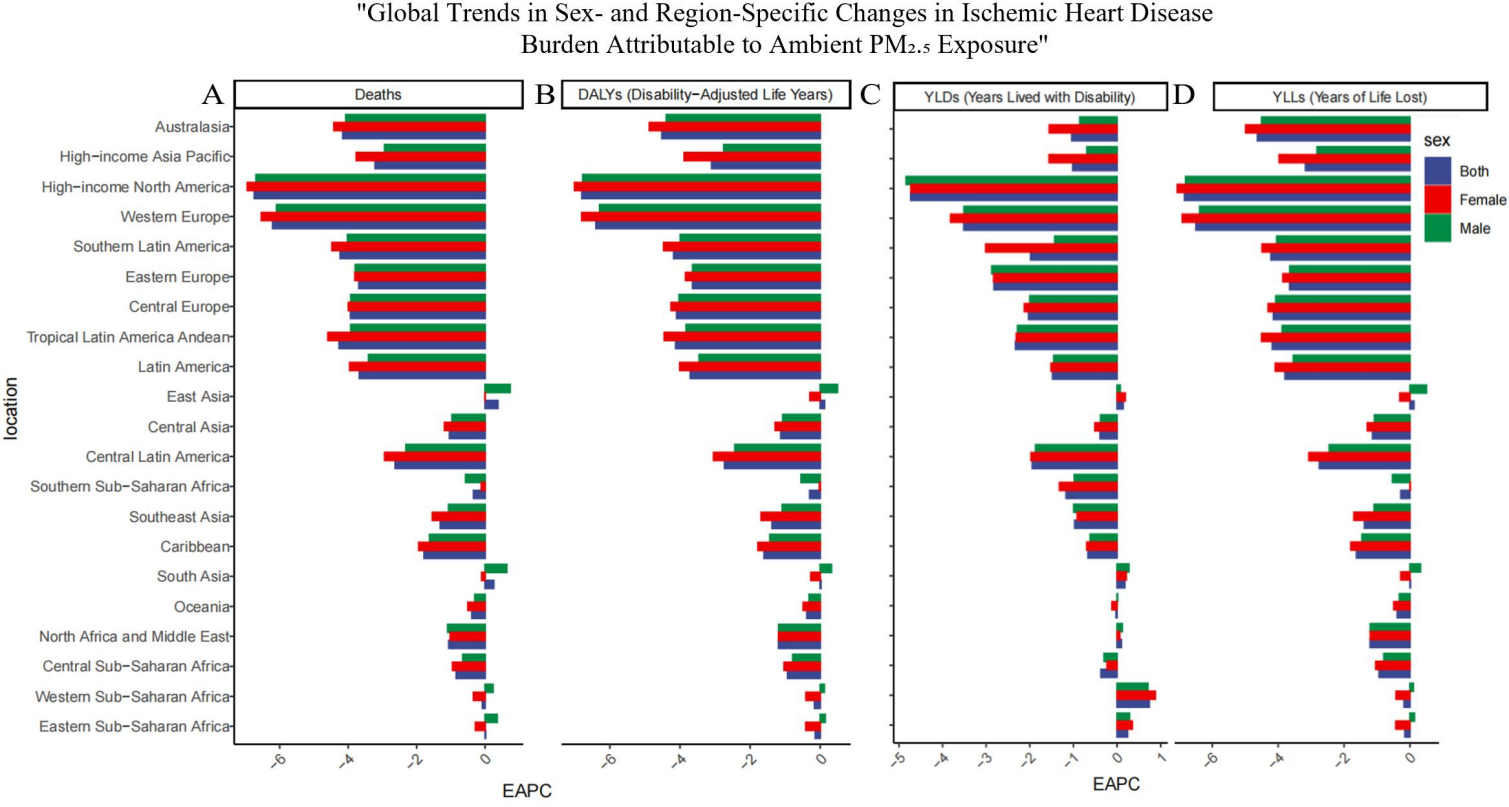
Regional distribution of temporal trends in ischemic heart disease (IHD) burden attributable to ambient PM_2.5_ pollution in 2021 across the 21 GBD regions. **(A)** Deaths, **(B)** Disability-adjusted life years (DALYs), **(C)** Years lived with disability (YLDs), and **(D)** Years of life lost (YLLs). Each panel presents the Estimated Annual Percentage Change (EAPC) in age-standardized rates per 100,000 population for the 21 Global Burden of Disease (GBD) regions. Positive EAPC values indicate increasing trends, while negative values indicate decreasing burdens of IHD linked to PM_2.5_ exposure.

Globally, particulate matter pollution progressively reduced impacts on cardiovascular disease in the elderly between 1990 and 2021. Estimated annual percentage changes (EAPC) and their 95% confidence intervals trend decisively negative, attesting to a sustained contraction in particulate-attributable ischemic heart disease (IHD). Over these three decades the age-standardized mortality rate (ASMR, per 100 000) receded from 296.414 (221.154–376.958) to 193.645 (143.121–243.059), while the age-standardized disability rate (ASDR) diminished from 4895.955 (3716.006–6 187.073) to 3242.217(2424.715–4048.509). This trend, however, varied significantly across the five SDI strata (Table 1). Using the middle-SDI group as the reference, higher-SDI regions show the lowest PM pollution levels, while lower-SDI areas carry the highest disease burden. Pockets of reversal persist: in low and low-middle SDI zones, older masculinities confront a rising burden despite the broader decline. Examination of the 21 GBD macro-regions reveals marked heterogeneity in trends (Figure 1). Positive EAPCs were observed in East Asia, South Asia, and both Western and Eastern Sub-Saharan Africa, reflecting rising risk in these areas. By contrast, most other regions displayed negative trajectories in ASMR and ASDR. High-income North America and Western Europe consistently recorded the lowest burden of particulate matter–attributable IHD across the study years.

**Table 1:** Global and regional burden of ischemic heart disease attributable to ambient PM_2.5_ exposure, 1990–2021.

Location	Cause	Rei	Sex	DALYs (Disability-Adjusted Life Years)_Number_ 1990_CI	DALYs (Disability-Adjusted Life Years)_Number_ 2021_CI	Deaths_ Number_ 1990_CI	Deaths_ Number_ 2021_CI	YLDs (Years Lived with Disability)_Number_ 1990_CI	YLDs (Years Lived with Disability)_Number_ 2021_CI	YLLs (Years of Life Lost)_Number_ 1990_CI	YLLs (Years o f Life Lost)_Number_ 2021_CI	Deaths_ASR_ 1990_CI	Deaths_ASR_ 2021_CI	DALYs (Disability-Adjusted Life Years)_ ASR_1990_CI	DALYs (Disability-Adjusted Life Years)_ASR_ 2021_CI	YLDs (Years Lived with Disability)_ASR_1990_CI	YLDs (Years Lived with Disability)_ASR_ 2021_CI	YLLs (Years of Life Lost)_ASR_1990_CI	YLLs (Years of Life Lost)_ASR_ 2021_CI	Deaths_ EAPC_95CI	DALYs (Disability- Adjusted Life Years)_ EAPC_95CI	YLDs (Years Lived with Disability)_ EAPC_95CI	YLLs (Years of Life Lost)_ EAPC_95CI	DALYs (Disability- Adjusted Life Years)_ number_ percent_change	Deaths_ number_ percent_change	YLDs (Years Lived with Disability) _number_ percent_change	YLLs (Years of Life Lost)_number_ percent_change
Global	Ischemic heart disease	Particulate matter pollution	Both	22,107,988 (1,6903,345 to 27,803,896)	34,564,173 (25,887,530 to 43,130,304)	1,221,853 (921,412 to 1,541,964)	2,005,125 (1,484,963 to 2,514,494)	400,588 (227,166 to 630,209)	777,285 (440,728 to 1,219,129)	21,707,400 (16,569,509 to 27,201,522)	33,786,889 (25,257,005 to 42,183,966)	296.414 (221.154 to 376.958)	193.645 (143.121 to 243.059)	4,895.955 (3,716.006 to 6,187.073)	3,242.217 (2,424.715 to 4,048.509)	86.78 (49.332 to 136.158)	72.131 (40.913 to 113.058)	4,809.175 (3,643.411 to 6,057.291)	3,170.087 (2,366.057 to 3,960.785)	−1.41 (−1.51 to −1.31)	−1.41 (−1.5 to −1.31)	−0.6 (−0.68 to −0.53)	−1.42 (−1.52 to −1.32)	56.342	64.105	94.036	55.647
High SDI	Ischemic heart disease	Particulate matter pollution	Both	4,390,759 (2,705,124 to 6,182,362)	2,040,754 (1,341,896 to 2,770,967)	271,777 (166,551 to 384,367)	137,862 (88,732 to 188,372)	81,321 (42,292 to 137,892)	79,453 (42,406 to 128,136)	4,309,438 (2,654,079 to 6,058,496)	1,961,301 (1,286,276 to 2,665,403)	192.919 (117.758 to 273.586)	43.567 (28.255 to 59.363)	3,058.692 (1,879.868 to 4,313.353)	689.945 (456.595 to 934.873)	56.042 (29.145 to 95.12)	27.673 (14.793 to 44.618)	3,002.649 (1,844.717 to 4,227.826)	662.272 (437.325 to 898.138)	−5.1 (−5.21 to −4.99)	−5.12 (−5.23 to −5.01)	−2.58 (−2.68 to −2.47)	−5.19 (−5.3 to −5.08)	−53.522	−49.274	−2.297	−54.488
High-middle SDI	Ischemic heart disease	Particulate matter pollution	Both	6,530,351 (4,660,856 to 8,614,951)	7,405,535 (5,275,594 to 9,674,445)	378,921 (268,219 to 500,825)	476,499 (338,048 to 621,357)	117,317 (65,196 to 184,272)	184,071 (104,392 to 288,201)	6,413,034 (4,572,934 to 8,453,220)	7,221,463 (5,132,103 to 9,439,931)	376.742 (264.377 to 499.804)	196.405 (139.206 to 256.076)	5,820.328 (4,129.53 to 7,695.422)	2,972.711 (2,116.555 to 3,882.584)	99.15 (55.218 to 155.337)	72.219 (40.929 to 112.965)	5,721.178 (4,055.805 to 7,559.658)	2,900.491 (2,059.936 to 3,790.46)	−2.32 (−2.57 to −2.07)	−2.45 (−2.73 to −2.17)	−1.04 (−1.17 to −0.9)	−2.48 (−2.76 to −2.19)	13.402	25.752	56.901	12.606
Low SDI	Ischemic heart disease	Particulate matter pollution	Both	1,504,487 (1,187,060 to 1,871,229)	3,095,481 (2,417,851 to 3,791,725)	72,857 (57,344 to 90,695)	159,504 (124,233 to 195665)	23,941 (13,792 to 37,764)	58,776 (33,816 to 91,924)	1,480,545 (1,166,160 to 1,845,387)	3,036,705 (2,369,682 to 3,723,829)	351.038 (274.943 to 436.616)	345.917 (268.265 to 424.894)	6,303.99 (4,962.199 to 7,833.275)	5,949.297 (4,635.251 to 7,291.377)	103.626 (60.054 to 162.459)	112.379 (65.034 to 174.817)	6,200.363 (4,872.09 to 7,720.695)	5,836.919 (4,543.129 to 7,162.585)	0.1 (−0.02 to 0.23)	−0.11 (−0.2 to −0.02)	0.3 (0.26 to 0.33)	−0.12 (−0.21 to −0.03)	105.75	118.927	145.504	105.107
Low-middle SDI	Ischemic heart disease	Particulate matter pollution	Both	4,322,387 (3,405,288 to 5,297,568)	9,761,841 (7,525,052 to 11,964,842)	215,084 (169,214 to 263,262)	513,785 (394,662 to 631,350)	69,744 (40,122 to 109,606)	175,731 (100,978 to 276,015)	4,252,643 (3,352,518 to 5,200,309)	9,586,110 (7,381,152 to 11,764,870)	370.363 (290.28 to 454.116)	345.249 (264.516 to 424.581)	6,610.626 (5,198.621 to 8,108.885)	6,016.446 (4,629.562 to 7,379.962)	110.265 (63.779 to 172.462)	109.006 (62.847 to 170.732)	6,500.361 (5,112.851 to 7,957.372)	5,907.44 (4,541.118 to 7,254.903)	−0.1 (−0.2 to 0)	−0.22 (−0.3 to −0.14)	0.02 (−0.05 to 0.09)	−0.22 (−0.31 to −0.14)	125.844	138.876	151.966	125.415
Middle SDI	Ischemic heart disease	Particulate matter pollution	Both	5,322,710 (4,168,043 to 6,534,869)	12,231,343 (9,080,868 to 15,415,001)	281,073 (219,059 to 344,897)	715,726 (527,950 to 901,298)	107,587 (61,646 to 169,311)	278,502 (159,055 to 440,722)	5,215,123 (4,077,742 to 6,402,485)	11,952,841 (8,851,617 to 15,054,643)	311.923 (241.513 to 383.191)	252.295 (185.77 to 317.797)	5,062.056 (3,947.455 to 6,216.481)	3,988.255 (2,956.564 to 5,025.445)	98.773 (57.008 to 154.654)	87.839 (50.256 to 138.841)	4,963.284 (3,864.005 to 6,094.85)	3,900.416 (2,883.706 to 4,912.707)	−0.53 (−0.71 to −0.34)	−0.67 (−0.84 to −0.5)	−0.28 (−0.41 to −0.15)	−0.68 (−0.85 to −0.51)	129.795	154.641	158.862	129.196
North Africa and Middle East	Ischemic heart disease	Particulate matter pollution	Both	1,736,865 (1,325,163 to 2,165,726)	3,258,683 (2,464,920 to 4,085,240)	90,807 (68,952 to 113,297)	179,872 (135,281 to 225,311)	19,432 (11,141 to 30,397)	54,638 (31,583 to 85,150)	1,717,433 (1,307,960 to 2,141,965)	3,204,045 (2,415,661 to 4,016,411)	581.913 (439.674 to 726.389)	416.772 (312.465 to 522.068)	9,835.741 (7,481.995 to 12,271.107)	6,838.191 (5,162.377 to 8,568.957)	109.349 (62.898 to 170.428)	111.862 (64.794 to 173.874)	9,726.392 (7,385.137 to 12,135.677)	6,726.329 (5,062.93 to 8,428.784)	−1.06 (−1.12 to −1)	−1.19 (−1.26 to −1.12)	0.1 (0.01 to 0.19)	−1.21 (−1.28 to −1.14)	87.619	98.082	181.175	86.56
South Asia	Ischemic heart disease	Particulate matter pollution	Both	4,146,959 (3,260,875 to 5,099,711)	11,140,635 (8,588,298 to 13,618,993)	199,158 (156,370 to 244,985)	579,956 (446,208 to 710,218)	67,933 (39,497 to 106,057)	203,371 (117,090 to 318,145)	4,079,026 (3,209,591 to 5,010,728)	10,937,264 (8,426,367 to 13,389,049)	366.536 (286.747 to 451.934)	376.955 (289.457 to 462.007)	6,751.518 (5,296.388 to 8,312.787)	6,599.063 (5,082.936 to 8,069.972)	117.748 (68.831 to 182.862)	121.83 (70.418 to 190.045)	6,633.77 (5,207.662 to 8,161.105)	6,477.233 (4,984.509 to 7,932.548)	0.25 (0.08 to 0.42)	0.02 (−0.11 to 0.14)	0.18 (0.05 to 0.3)	0.01(−0.11 to 0.14)	168.646	191.204	199.37	168.134

### 3.2 Trends in national burden of elderly IHD due to ambient particulate matter pollution, 1990 to 2021

**Figure 2:**
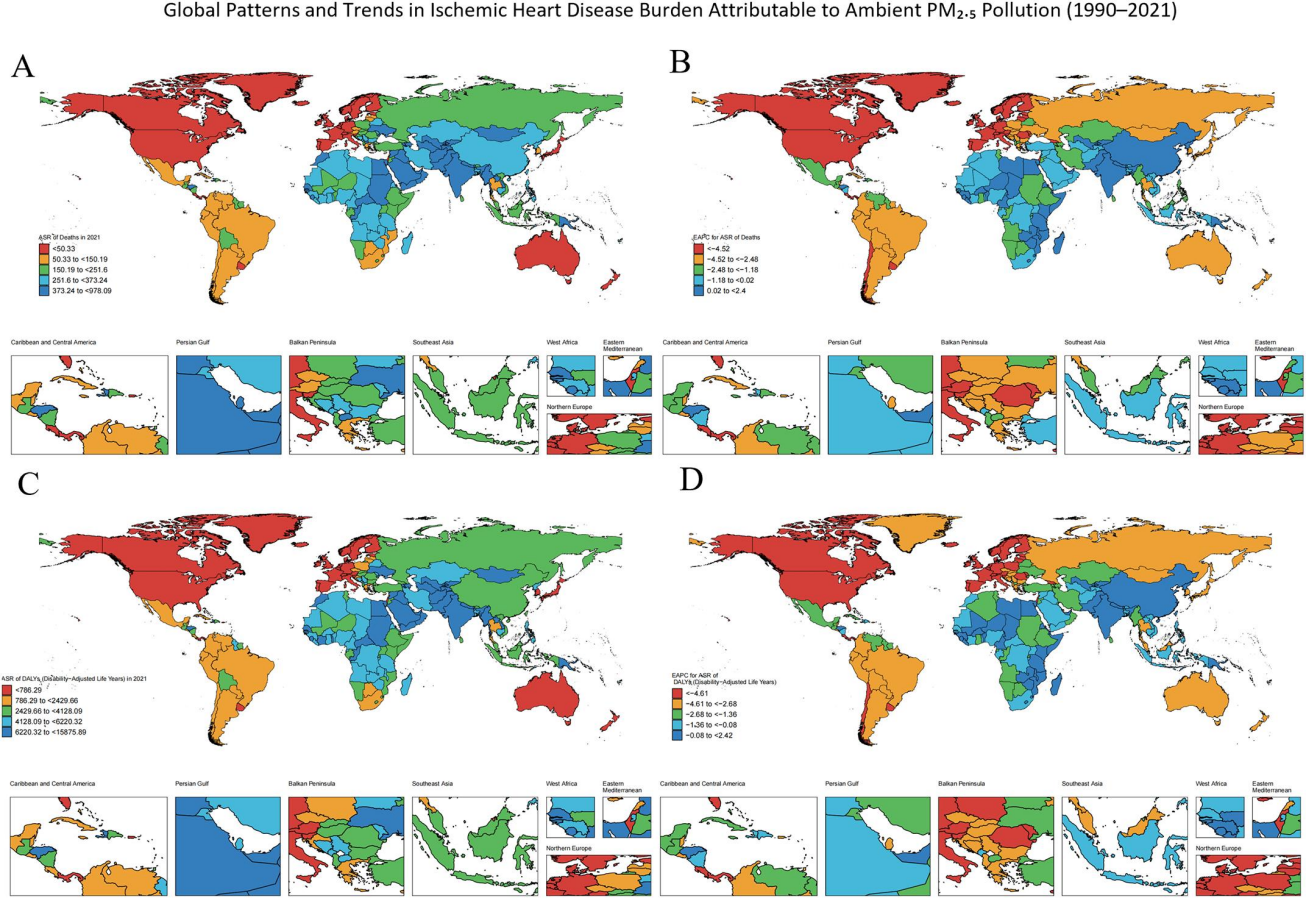
Global distribution of temporal trends in ischemic heart disease (IHD) burden attributable to ambient PM₂.₅ pollution from 2021. **(A)** Deaths, **(B)** Estimated Annual Percentage Change (EAPC) in deaths from 1990 to 2021, **(C)** Disability-adjusted life years (DALYs), **(D)** EAPC in DALYs from 1990 to 2021.Each panel shows age-standardized rates per 100,000 population at the national level.

Using data from the 204 countries and territories included in the GBD archive, we generated a choropleth world map (Figures 2A,B) to illustrate the global distribution of ischemic heart disease (IHD) attributable to ambient particulate matter pollution. In 2021, the highest burdens were observed in Kyrgyzstan, Pakistan, Guinea-Bissau, and The Gambia, where the age-standardized mortality rate (ASMR) exceeded 400 per 100,000 population and the age-standardized disability rate (ASDR) surpassed 5,000 per 100,000. The detailed numerical estimates supporting these visualizations are provided in the Supplementary Tables S1.

From 1990 to 2021, the majority of the 204 territorial units experienced a reduction in ischemic heart disease (IHD) attributable to particulate matter exposure. Nevertheless, this decline was heterogeneous. Lesotho, Zimbabwe, and Kenya recorded the highest positive estimated annual percentage changes (EAPCs) for both age-standardized mortality rates (ASMR) and age-standardized disability rates (ASDR), reflecting a notable increase in burden contrary to the broader downward trend. A full roster of EAPC values for every polity is likewise archived in the Supplementary Tables S1.

### 3.3 Disparities in IHD burden due to ambient particulate matter pollution by age and sex in older adults

**Figure 3:**
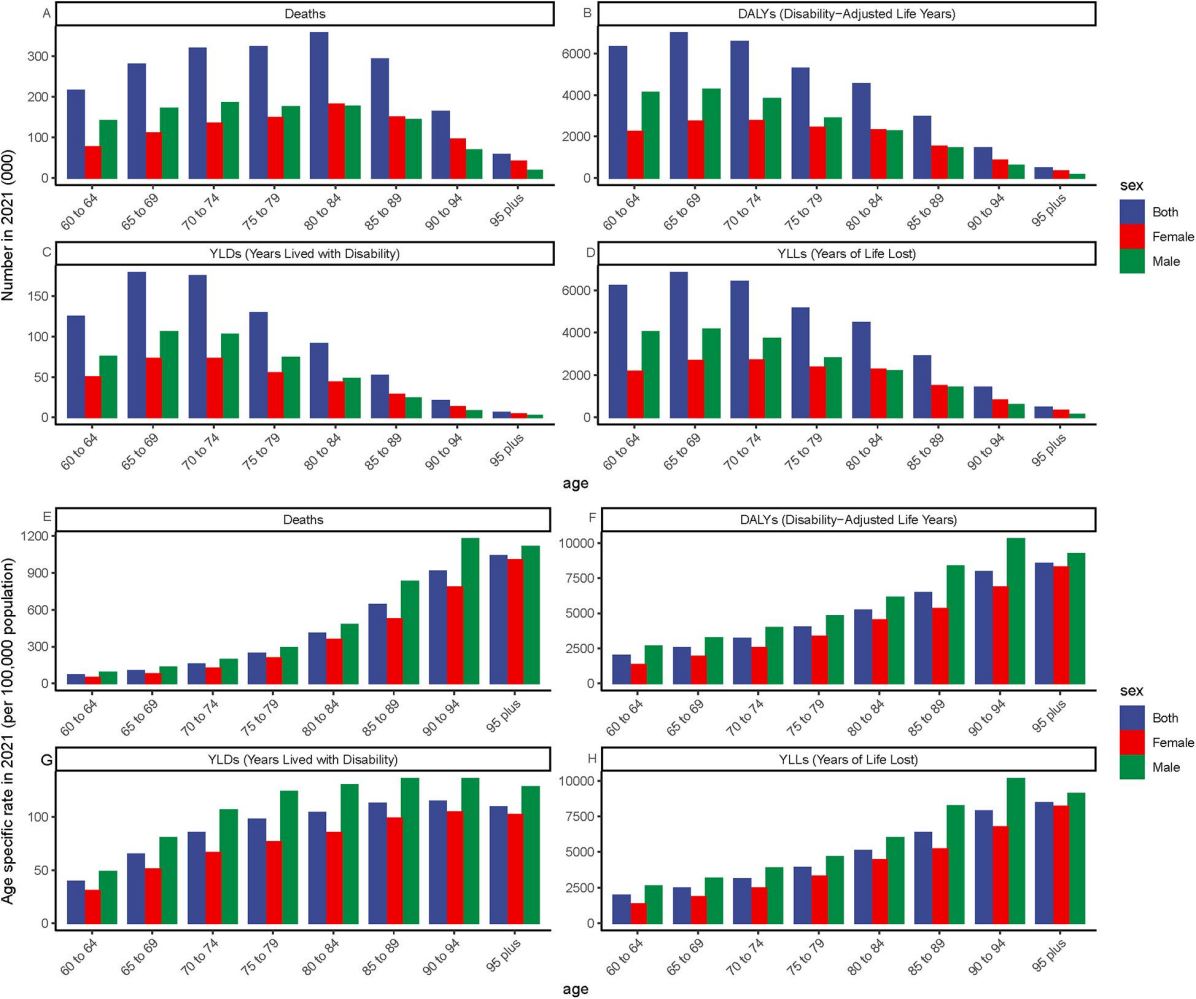
Global burden of ischemic heart disease (IHD) attributable to ambient PM₂.₅ pollution among older adults in 2021, stratified by age. **(A–D)** Absolute number of cases: Deaths, DALYs, YLDs, and YLLs. **(E–H)** Age-specific rates per 100,000 population: Deaths, DALYs, YLDs, and YLLs. Panels reflect the distribution and intensity of IHD burden across age groups in older populations.

**Figure 4:**
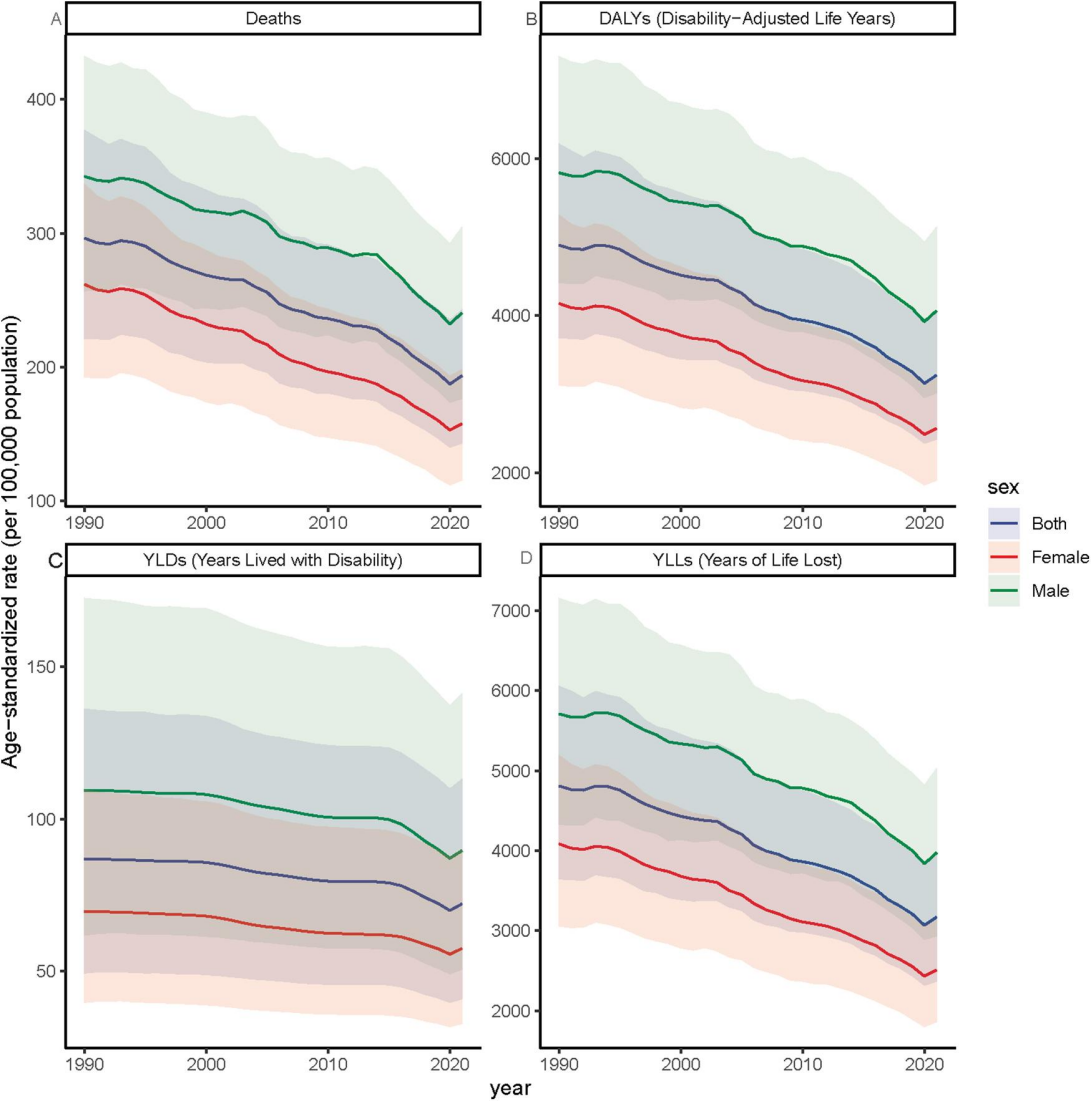
Trends in age-standardized rates of ischemic heart disease (IHD) attributable to ambient PM_2.5_ pollution among older adults, by sex, from 1990 to 2021.Age-standardized rates per 100,000 population are shown for **(A)** deaths, **(B)** DALYs, **(C)** YLDs, and **(D)** YLLs, disaggregated by males, females, and the total elderly population (aged ≥60 years). The figure illustrates long-term trends in IHD burden due to PM₂.₅ exposure across sex groups.

**Figure 5:**
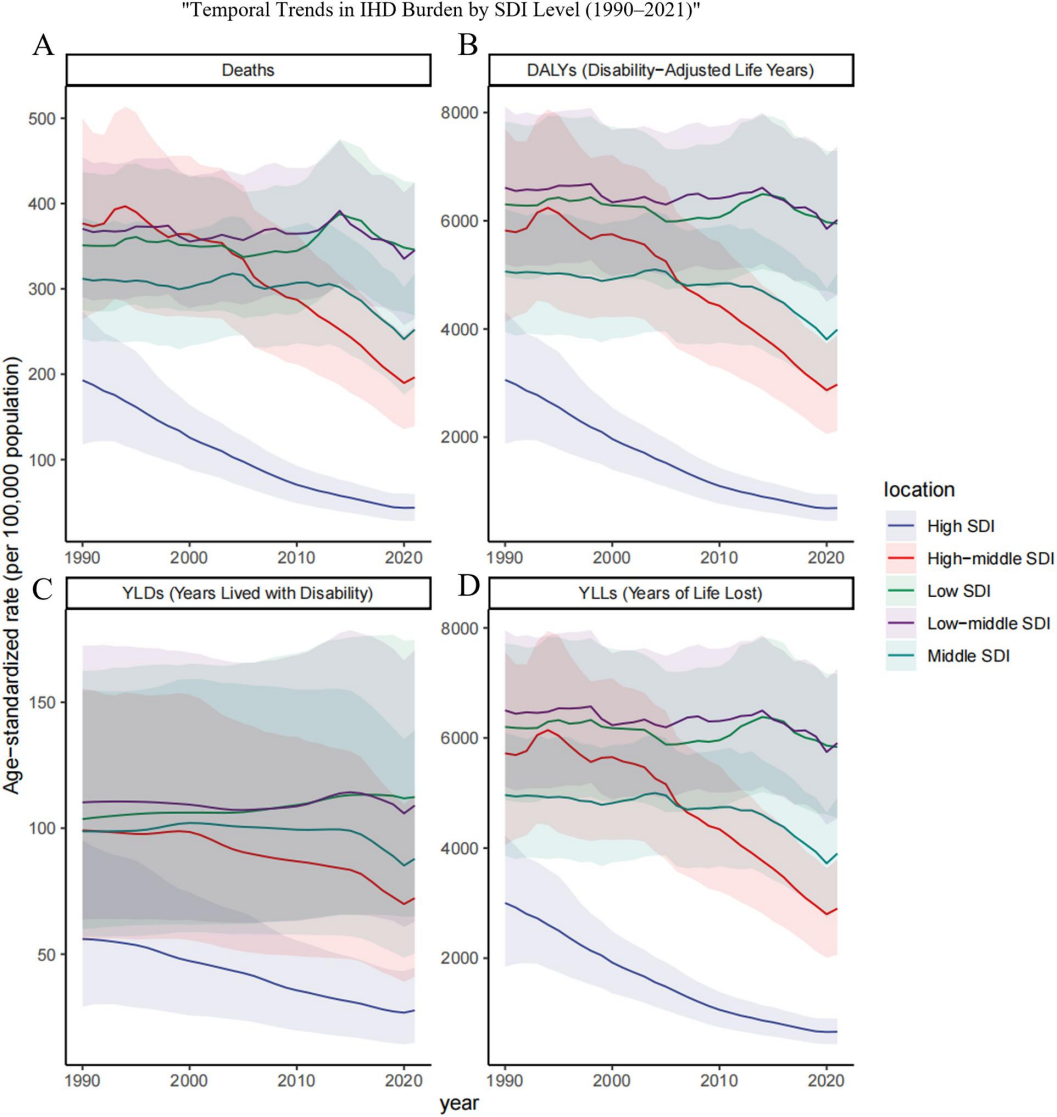
Trends in age-standardized rates of ischemic heart disease (IHD) attributable to ambient PM_2.5_ pollution among older adults across different SDI regions from 1990 to 2021.Age-standardized rates per 100,000 population are presented for **(A)** deaths, **(B)** DALYs, **(C)** YLDs, and **(D)** YLLs in five sociodemographic index (SDI) regions: low, low-middle, middle, high-middle, and high. The figure highlights disparities in the temporal evolution of IHD burden due to PM_2.5_ exposure among elderly populations across varying levels of socioeconomic development.

Globally, (Figure 3) demonstrates the impact of ambient particulate matter pollution on ischemic heart disease (IHD) in 2021. This is assessed through mortality rates, disability-adjusted life year (DALY) rates, and their estimated annual percentage changes (EAPCs) across different age and sex categories. When considering age solely from a numerical perspective, mortality rates and disability-adjusted life year (DALY) rates among individuals aged 60 years and older are generally highest between the ages of 65 and 85 (Figures 3A–D). Age-specific rates show a steady increase with advancing age, peaking among those aged 95 years and older. In this oldest age group, the burden of ischemic heart disease (IHD) attributable to ambient particulate matter pollution is the most pronounced, indicating that very elderly individuals are particularly vulnerable to the cardiovascular effects of environmental exposure (Figures 3E–H). In terms of gender, older men generally experienced a higher IHD burden due to ambient particulate matter pollution compared to women, with the largest difference observed in the 85–94 age group. A comparison between 1990 and 2021 reveals a general reduction in disease burden across almost all age and sex groups by 2021 (Table 2). Nevertheless, in areas classified as low and low-middle SDI, a rising pattern persisted across different age groups, most notably among people aged 80 and above, who faced the highest ischemic heart disease (IHD) burden due to particulate pollution. Within these regions, older men consistently experienced a higher burden compared to women. Remarkably, in the low-middle SDI region, although the overall burden for individuals aged 80 and older continued to increase, the burden among females in this age group showed a declining trend. Furthermore, at the national level, we examined the correlation between gender and the EAPC values of disease burden indicators across countries and territories, and found a statistically significant association (Figures 4, 5).

**Table 2:** Global age- and sex-specific burden of ischemic heart disease attributable to ambient PM_2.5_ exposure, 1990–2021.

Location	cause	sex	age	DALYs (Disability-Adjusted Life Years)_Number_1990_CI	DALYs (Disability-Adjusted Life Years)_Number_2021_CI	Deaths_Number_1990_CI	Deaths_Number_2021_CI	YLDs (Years Lived with Disability)_Number_1990_CI	YLDs (Years Lived with Disability)_Number_2021_CI	YLLs (Years of Life Lost)_Number_1990_CI	YLLs (Years of Life Lost)_Number_2021_CI	Deaths_ASR_1990_CI	Deaths_ASR_2021_CI	DALYs (Disability-Adjusted Life Years)_ASR_ 1990_CI	DALYs (Disability-Adjusted Life Years)_ASR_2021_CI	YLDs (Years Lived with Disability)_ASR_ 1990_CI	YLDs (Years Lived with Disability)_ASR_ 2021_CI	YLLs (Years of Life Lost)_ASR_1990_CI	YLLs (Years of Life Lost)_ASR_2021_CI	Deaths_EAPC_95CI	DALYs (Disability-Adjusted Life Years)_EAPC_95CI	YLDs (Years Lived with Disability)_EAPC_95CI	YLLs (Years of Life Lost)_EAPC_95CI	DALYs (Disability-Adjusted Life Years)_number_percent_change	Deaths_number_percent_change	YLDs (Years Lived with Disability)_number_percent_change	YLLs (Years of Life Lost)_number_percent_change
Global	Ischemic heart disease	Female	60 to 64	1,738,308 (1,350,831 to 2,154,278)	2,219,247 (1,684,114 to 2,737,869)	59,241 (45,789 to 73,217)	75,149 (57,027 to 92,813)	29,116 (16,500 to 48,495)	49,805 (28,255 to 82,019)	1,709,192 (1,321,104 to 2,112,374)	2,169,442 (1,646,325 to 2,679,401)	72.19 (55.797 to 89.22)	45.68 (34.665 to 56.417)	2,118.257 (1,646.088 to 2,625.148)	1,348.996 (1,023.71 to 1,664.248)	35.48 (20.106 to 59.095)	30.274 (17.175 to 49.856)	2,082.778 (1,609.863 to 2,574.084)	1,318.722 (1,000.739 to 1,628.707)	−1.69 (−1.8 to −1.58)	−1.66 (−1.77 to −1.56)	−0.56 (−0.63 to −0.49)	−1.69 (−1.79 to −1.58)	27.667	26.853	71.057	26.928
Global	Ischemic heart disease	Female	65 to 69	1,938,954 (1,507,020 to 2,406,338)	2,734,613 (2,058,244 to 3,385,756)	78,168 (60,665 to 97,185)	109,580 (82,568 to 136,071)	38,914 (22,416 to 61,434)	72,915 (42,235 to 114,385)	1,900,040 (1,474,713 to 2,362,613)	2,661,698 (2,005,392 to 3,305,508)	117.939 (91.531 to 146.632)	76.092 (57.336 to 94.488)	2,925.476 (2,273.777 to 3,630.66)	1,898.919 (1,429.247 to 2,351.073)	58.713 (33.822 to 92.691)	50.632 (29.328 to 79.429)	2,866.763 (2,225.034 to 3,564.688)	1,848.287 (1,392.547 to 2,295.35)	−1.65 (−1.78 to −1.51)	−1.62 (−1.75 to −1.49)	−0.61 (−0.67 to −0.54)	−1.65 (−1.78 to −1.51)	41.035	40.185	87.375	40.086
Global	Ischemic heart disease	Female	70 to 74	1,863,901 (1,440,094 to 2,326,377)	2,756,908 (2,081,393 to 3,422,646)	91,450 (70,579 to 114,365)	134,068 (101,476 to 167,085)	37,087 (20,653 to 57,494)	72,456 (41,117 to 112,278)	1,826,813 (1,409,970 to 2,284,489)	2,684,451 (2,031,771 to 3,345,655)	194.398 (150.032 to 243.109)	122.495 (92.716 to 152.661)	3,962.151 (3,061.252 to 4,945.252)	2,518.925 (1,901.722 to 3,127.195)	78.838 (43.902 to 122.218)	66.202 (37.567 to 102.586)	3,883.314 (2,997.217 to 4,856.209)	2,452.723 (1,856.384 to 3,056.85)	−1.57 (−1.75 to −1.39)	−1.55 (−1.72 to −1.38)	−0.61 (−0.7 to −0.51)	−1.58 (−1.75 to −1.4)	47.911	46.603	95.368	46.947
Global	Ischemic heart disease	Female	75 to 79	1,998,088 (1,504,319 to 2,543,893)	2,418,891 (1,811,557 to 3,004,877)	123,302 (92,838 to 156,864)	148,189 (111,380 to 184,065)	33,611 (18,738 to 52,627)	55,177 (30,307 to 84,068)	1,964,478 (1,479,559 to 2,498,515)	2,363,714 (1,776,904 to 2,935,769)	339.463 (255.594 to 431.863)	205.539 (154.485 to 255.3)	5,500.938 (4,141.542 to 7,003.593)	3,355.019 (2,512.643 to 4,167.786)	92.533 (51.589 to 144.888)	76.531 (42.036 to 116.603)	5,408.405 (4,073.376 to 6,878.662)	3,278.488 (2,464.579 to 4,071.933)	−1.51 (−1.62 to −1.39)	−1.5 (−1.61 to −1.38)	−0.56 (−0.64 to −0.47)	−1.52 (−1.63 to −1.4)	21.06	20.184	64.164	20.323
Global	Ischemic heart disease	Female	80 to 84	1,597,134 (1,167,059 to 2,050,630)	2,302,680 (1,678,150 to 2,877,919)	125,950 (91,543 to 162,057)	181,242 (131,380 to 226,041)	23,608 (13,614 to 35,953)	43,467 (24,260 to 66,952)	1,573,526 (1,144,097 to 2,024,220)	2,259,213 (1,637,896 to 2,817,105)	570.106 (414.363 to 733.54)	355.856 (257.956 to 443.816)	7,229.306 (5,282.605 to 9,282.023)	4,521.158 (3,294.935 to 5,650.602)	106.858 (61.623 to 162.74)	85.345 (47.634 to 131.456)	7,122.448 (5,178.671 to 9,162.482)	4,435.813 (3,215.9 to 5,531.198)	−1.48 (−1.6 to −1.35)	−1.47 (−1.59 to −1.34)	−0.69 (−0.75 to −0.63)	−1.48 (−1.6 to −1.36)	44.176	43.9	84.12	43.576
Global	Ischemic heart disease	Female	85 to 89	934,361 (664,012 to 1,223,495)	1,510,206 (1,079,368 to 1,934,378)	92,813 (65,913 to 121,565)	149,532 (106,764 to 191,612)	12,876 (7,741 to 19,928)	28,107 (17,193 to 43,831)	921,485 (654,520 to 1,206,390)	1,482,099 (1,058,534 to 1,898,853)	923.787 (656.05 to 1,209.962)	525.244 (375.016 to 673.053)	9,299.887 (6,609.047 to 12,177.7)	5,304.715 (3,791.364 to 6,794.65)	128.154 (77.046 to 198.35)	98.728 (60.391 to 153.961)	9,171.734 (6,514.573 to 12,007.452)	5,205.987 (3,718.181 to 6,669.865)	−1.83 (−1.93 to −1.74)	−1.82 (−1.92 to −1.73)	−0.97 (−1.01 to −0.93)	−1.84 (−1.94 to −1.74)	61.63	61.111	118.29	60.838
Global	Ischemic heart disease	Female	90 to 94	370,289 (253,032 to 498,187)	824,989 (574,945 to 1,070,206)	42,458 (29,023 to 57,164)	94,304 (65,523 to 122,045)	4,292 (2,552 to 6,773)	12,594 (7,522 to 19,579)	365,997 (250,226 to 492,731)	812,395 (564,488 to 1,051,342)	1,402.996 (959.049 to 1,888.967)	781.901 (543.268 to 1,011.906)	12,235.995 (8,361.301 to 16,462.329)	6,840.207 (4,767.022 to 8,873.367)	141.819 (84.346 to 223.801)	104.416 (62.369 to 162.337)	12,094.176 (8,268.589 to 16,282.013)	6,735.79 (4,680.323 to 8,716.966)	−2.03 (−2.12 to −1.94)	−2.02 (−2.11 to −1.93)	−1.2 (−1.25 to −1.15)	−2.03 (−2.12 to −1.94)	122.796	122.111	193.43	121.968
Global	Ischemic heart disease	Female	95 plus	125,207 (81,563 to 174,752)	325,657 (218,699 to 430,913)	15,232 (9,915 to 21,269)	39,597 (26,407 to 52,440)	1,156 (648 to 1,828)	4,024 (2,250 to 6,221)	124,051 (80,745 to 173,042)	321,633 (214,688 to 425,654)	2,009.852 (1,308.358 to 2,806.531)	1,005.454 (670.515 to 1,331.544)	16,521.248 (10,762.287 to 23,058.723)	8,269.044 (5,553.186 to 10,941.696)	152.595 (85.445 to 241.257)	102.168 (57.134 to 157.964)	16,368.653 (10,654.437 to 22,833.109)	8,166.876 (5,451.328 to 10,808.147)	−2.39 (−2.5 to −2.28)	−2.41 (−2.52 to −2.3)	−1.4 (−1.46 to −1.34)	−2.42 (−2.53 to −2.31)	160.095	159.959	248.097	159.275
Global	Ischemic heart disease	Male	60 to 64	3,020,168 (2,346,401 to 3,744,807)	4,113,019 (3,112,465 to 5,130,235)	102,944 (80,229 to 127,716)	139,756 (104,760 to 174,971)	46,007 (26,363 to 76,718)	75,294 (43,067 to 124,220)	2,974,162 (2,317,600 to 3,689,724)	4,037,725 (3,026,794 to 5,054,736)	131.062 (102.143 to 162.6)	89.854 (67.353 to 112.495)	3,845.09 (2,987.291 to 4,767.655)	2,644.4 (2,001.11 to 3,298.403)	58.573 (33.563 to 97.672)	48.409 (27.689 to 79.865)	3,786.517 (2,950.623 to 4,697.527)	2,595.991 (1,946.029 to 3,249.862)	−1.43 (−1.52 to −1.34)	−1.42 (−1.51 to −1.32)	−0.62 (−0.71 to −0.54)	−1.43 (−1.52 to −1.34)	36.185	35.759	63.658	35.76
Global	Ischemic heart disease	Male	65 to 69	2,707,551 (2,079,504 to 3,369,611)	4,257,788 (3,181,424 to 5,372,401)	108,909 (83,466 to 135,100)	170,623 (126,941 to 215,680)	55,119 (31,203 to 87,207)	106,044 (61,214 to 168,200)	2,652,432 (2,033,131 to 3,290,043)	4,151,744 (3,089,013 to 5,248,273)	189.965 (145.586 to 235.648)	129.423 (96.289 to 163.601)	4,722.647 (3,627.176 to 5,877.446)	3,229.677 (2,413.218 to 4,075.149)	96.142 (54.426 to 152.11)	80.438 (46.433 to 127.585)	4,626.505 (3,546.289 to 5,738.659)	3,149.239 (2,343.122 to 3,980.994)	−1.48 (−1.61 to −1.34)	−1.46 (−1.59 to −1.33)	−0.62 (−0.7 to −0.53)	−1.48 (−1.61 to −1.34)	57.256	56.666	92.391	56.526
Global	Ischemic heart disease	Male	70 to 74	2,120,747 (1,638,751 to 2,629,603)	3,816,052 (2,834,677 to 4,820,849)	103,456 (79,395 to 128,269)	185,289 (137,184 to 234,744)	48,415 (26,750 to 75,208)	102,435 (56,634 to 161,046)	2,072,332 (1,590,445 to 2,569,226)	3,713,617 (2,749,816 to 4,704,500)	275.013 (211.053 to 340.973)	192.225 (142.32 to 243.532)	5,637.508 (4,356.236 to 6,990.183)	3,958.913 (2,940.798 to 5,001.327)	128.699 (71.109 to 199.924)	106.27 (58.755 to 167.075)	5,508.809 (4,227.825 to 6,829.686)	3,852.643 (2,852.76 to 4,880.621)	−1.31 (−1.47 to −1.15)	−1.3 (−1.45 to −1.15)	−0.58 (−0.69 to −0.47)	−1.32 (−1.47 to −1.16)	79.939	79.099	111.577	79.2
Global	Ischemic heart disease	Male	75 to 79	1,782,392 (1,345,728 to 2,240,486)	2,862,696 (2,115,925 to 3,632,681)	109,135 (82,154 to 137,222)	174,176 (127,820 to 221,519)	37,208 (20,465 to 58,339)	73,910 (40,807 to 112,628)	1,745,184 (1,314,068 to 2,193,723)	2,788,786 (2,046,885 to 3,546,467)	432.51 (325.582 to 543.824)	291.329 (213.793 to 370.515)	7,063.781 (5,333.244 to 8,879.25)	4,788.179 (3,539.121 to 6,076.066)	147.459 (81.106 to 231.204)	123.622 (68.254 to 188.383)	6,916.322 (5,207.771 to 8,693.921)	4,664.557 (3,423.645 to 5,931.864)	−1.16 (−1.3 to −1.02)	−1.16 (−1.3 to −1.01)	−0.49 (−0.6 to −0.38)	−1.17 (−1.31 to −1.03)	60.61	59.597	98.64	59.799
Global	Ischemic heart disease	Male	80 to 84	1,149,859 (853,609 to 1,459,007)	2,242,185 (1,639,762 to 2,852,127)	89,912 (66,609 to 113,836)	175,501 (127,385 to 223,700)	21,447 (12,124 to 32,899)	47,727 (26,544 to 74,978)	1,128,412 (836,026 to 1,428,321)	2,194,458 (1,592,987 to 2,796,391)	676.875 (501.441 to 856.977)	478.831 (347.554 to 610.337)	8,656.334 (6,426.116 to 10,983.657)	6,117.521 (4,473.885 to 7,781.67)	161.455 (91.274 to 247.669)	130.218 (72.421 to 204.567)	8,494.878 (6,293.743 to 10,752.648)	5,987.303 (4,346.266 to 7,629.602)	−0.99 (−1.12 to −0.85)	−0.99 (−1.12 to −0.86)	−0.59 (−0.68 to −0.49)	−1 (−1.13 to −0.86)	94.997	95.192	122.535	94.473
Global	Ischemic heart disease	Male	85 to 89	538,806 (397,873 to 689,771)	1,440,790 (1,054,876 to 1,824,229)	53,175 (39,214 to 68,102)	142,872 (104,230 to 180,449)	8,874 (5,342 to 13,617)	23,464 (13,944 to 36,062)	529,932 (390,782 to 678,588)	1,417,325 (1,034,037 to 1,790,124)	1,050.047 (774.358 to 1,344.812)	828.118 (604.14 to 1,045.917)	10,639.806 (7,856.798 to 13,620.912)	8,351.116 (6,114.278 to 10,573.611)	175.239 (105.488 to 268.904)	136.003 (80.825 to 209.023)	10,464.568 (7,716.768 to 13,400.069)	8,215.112 (5,993.492 to 10,375.93)	−0.68 (−0.83 to −0.53)	−0.7 (−0.85 to −0.55)	−0.85 (−0.9 to −0.8)	−0.7 (−0.84 to −0.55)	167.404	168.683	164.413	167.454
Global	Ischemic heart disease	Male	90 to 94	178,680 (129,878 to 231,189)	598,949 (431,098 to 763,147)	20,431 (14,732 to 26,423)	68,436 (49,146 to 87,120)	2,359 (1,393 to 3,676)	7,928 (4,700 to 12,154)	176,321 (127,158 to 228,004)	591,021 (424,394 to 752,434)	1,622.859 (1,170.17 to 2,098.792)	1,174.158 (843.206 to 1,494.717)	14,192.629 (10,316.252 to 18,363.502)	10,276.215 (7,396.382 to 13,093.371)	187.383 (110.61 to 291.955)	136.023 (80.632 to 208.523)	14,005.246 (10,100.212 to 18,110.525)	10,140.192 (7,281.358 to 12,909.572)	−0.87 (−1.05 to −0.68)	−0.87 (−1.05 to −0.68)	−1.15 (−1.23 to −1.07)	−0.86 (−1.05 to −0.67)	235.208	234.962	236.075	235.196
Global	Ischemic heart disease	Male	95 plus	43,543 (30,227 to 57,941)	139,505 (99,363 to 179,433)	5,276 (3,658 to 7,037)	16,813 (11,970 to 21,663)	500 (282 to 790)	1,938 (1,086 to 3,013)	43,043 (29,851 to 57,409)	137,567 (98,015 to 177,141)	2,027.359 (1,405.826 to 2,704.081)	1,111.911 (791.657 to 1,432.722)	16,732.517 (11,615.412 to 22,265.055)	9,226.262 (6,571.462 to 11,866.919)	192.28 (108.295 to 303.493)	128.188 (71.791 to 199.293)	16,540.238 (11,471.083 to 22,060.815)	9,098.074 (6,482.283 to 11,715.376)	−1.82 (−2.02 to −1.63)	−1.83 (−2.03 to −1.64)	−1.38 (−1.53 to −1.24)	−1.84 (−2.04 to −1.64)	220.384	218.669	287.6	219.604

### 3.4 Relationship between SDI and the burden and temporal trends of elderly IHD attributable to ambient particulate matter pollution

**Figure 6:**
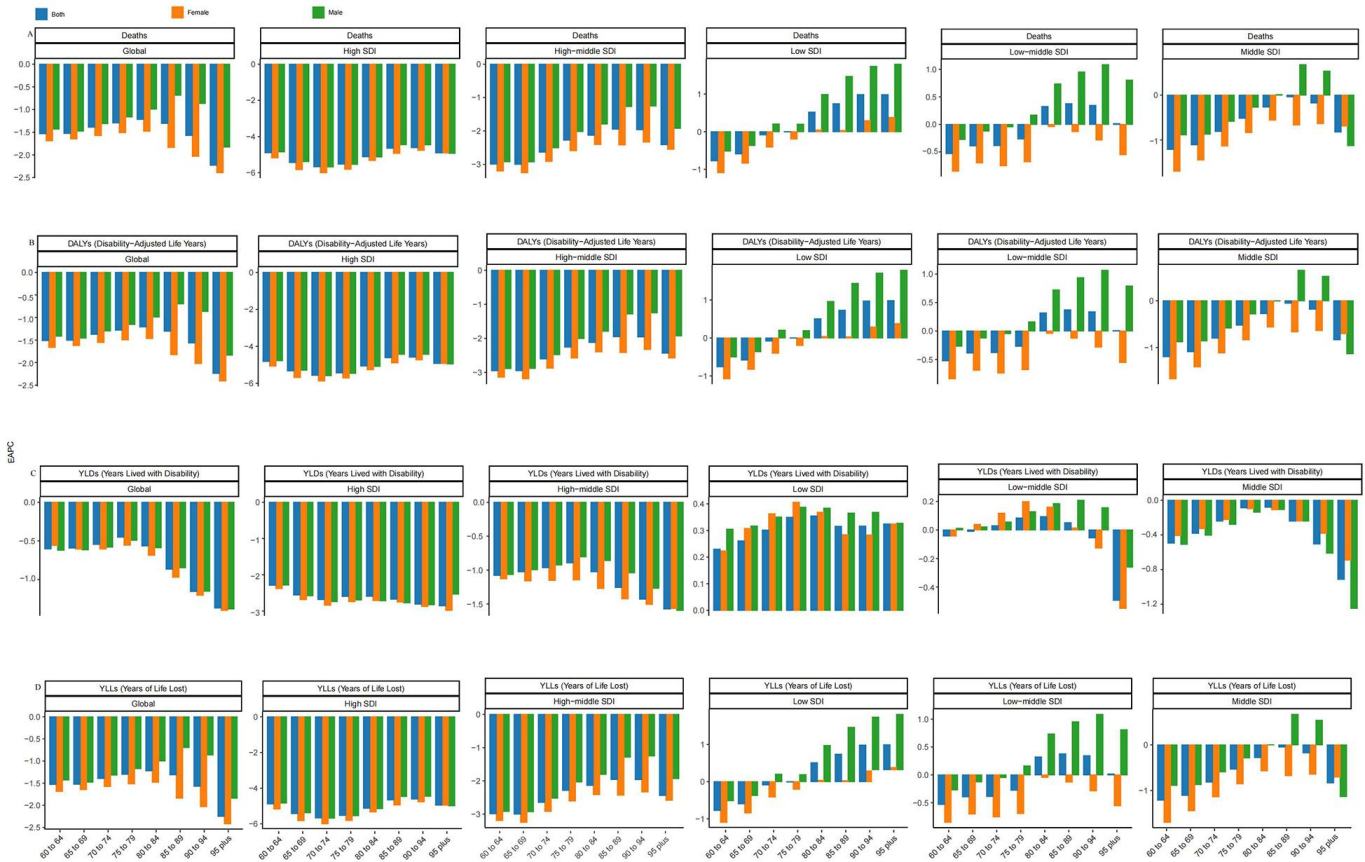
EAPC of ischemic heart disease (IHD) burden attributable to ambient PM_2.5_ pollution among older adults across age groups and SDI regions. **(A)** Deaths, **(B)** DALYs, **(C)** YLDs, and **(D)** YLLs. Each panel shows the Estimated Annual Percentage Change (EAPC) of age-standardized rates from 1990 to 2021 across different elderly age groups within six SDI levels (low, low-middle, middle, high-middle, high, and global). The figure illustrates disparities in IHD burden trends by age and regional development status.

Situated within a broader political-economic landscape, the particulate-related burden of ischemic heart disease (IHD) appears to track regional gradients of social development. To probe this relationship, we arrayed standardized mortality, disability, and trajectory metrics against the Socio-demographic Index (SDI) for the twenty-one GBD macro-regions over the 1990–2021 timespan, and, in parallel, mapped 2021 data for 204 countries and territories to capture present-day differentials (Figures 2C–6). Across both scales, a pronounced inverse association emerged (*P* < 0.05): higher SDI scores consistently coincided with lower IHD burdens, and the estimated annual percentage change (EAPC) likewise declined as SDI rose. Locally weighted regression lines, employed to detect departures from this general pattern, spotlighted several outliers whose observed burdens substantially exceeded SDI-based expectations. Most conspicuous were Andean Latin America and Southern Sub-Saharan Africa, where the particulate-attributable IHD load remains markedly higher than the developmental profile alone would predict.

### 3.5 Sex-based forecast of ambient PM–attributable IHD burden in older adults: a global outlook to 2040

**Figure 7:**
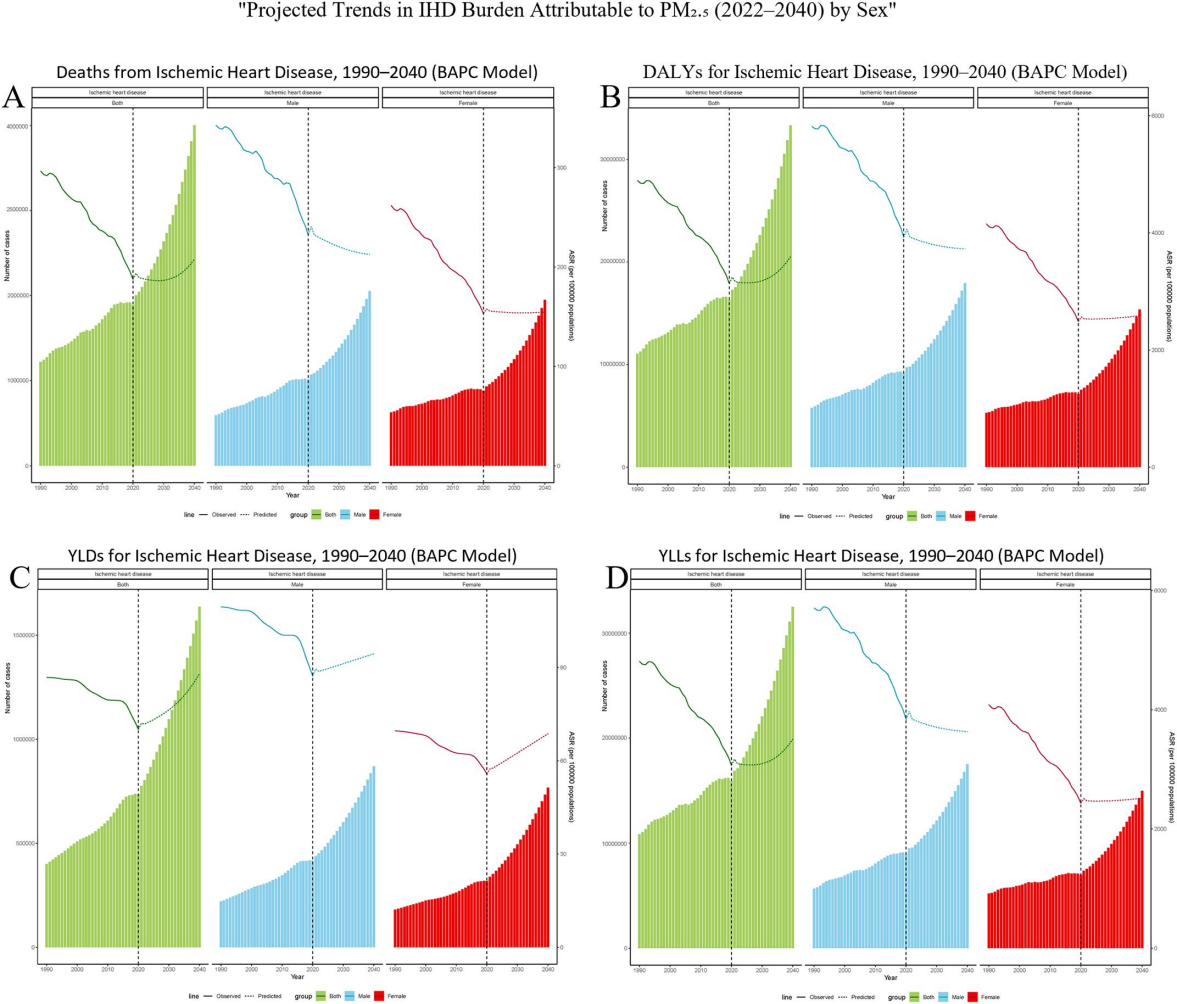
Bayesian projections of ischemic heart disease (IHD) burden attributable to ambient PM₂.₅ pollution among older adults by sex from 1990 to 2040. **(A)** Deaths, **(B)** DALYs, **(C)** YLDs, and **(D)** YLLs. Each panel presents age-standardized rates per 100,000 population, separately for males, females, and the total elderly population. Solid lines represent observed trends from 1990 to 2021; dashed lines indicate projected estimates from 2022 to 2040, based on a Bayesian age–period–cohort (BAPC) model.

Globally, a Bayesian age-period-cohort (BAPC) model was developed using data from 1990 to 2021. This model aimed to predict sex-specific trends in the burden of ischemic heart disease (IHD) caused by ambient particulate matter pollution among adults aged 60 and above for the years 2022 to 2040 (Figure 7). The results suggest that for both genders, the age-standardized mortality rate (ASMR) and age-standardized disability rate (ASDR) are anticipated to decrease during the early part of the forecast period, followed by a possible resurgence around 2035, potentially surpassing current levels. Conversely, the estimated annual percentage change (EAPC) for years lived with disability (YLDs) is expected to rise consistently throughout this timeframe. When analyzed by gender, ASMR and ASDR among males are projected to keep declining, while these rates among females are likely to remain fairly constant. Nevertheless, the upward trend in YLD-related EAPC is expected to persist in both males and females over time.

## 4 Discussion

During the past decade, recognition of the adverse health consequences of outdoor particulate matter (PM) exposure has increased considerably, with fine particles such as PM₂.₅ attracting particular concern. Older adults represent a population especially sensitive to such exposures due to age-related physiological decline. Ischemic heart disease (IHD), a leading global cause of mortality, arises from multiple interacting risk factors, and growing evidence implicates ambient air pollution as a major contributor to cardiovascular risk in the elderly. Drawing on estimates from the Global Burden of Disease (GBD) 2021, the present study evaluates temporal patterns in the burden of IHD attributable to PM among older populations.

Global and SDI-stratified analyses reveal an overall reduction in ischemic heart disease (IHD) linked to ambient particulate matter (PM) exposure, yet this decline is not uniform. Of the 204 countries and territories examined, 38 exhibited either stable or increasing burdens. Such heterogeneity may be attributed to uneven implementation and effectiveness of environmental regulations and public health initiatives over the past decades. The obstacles are especially acute in lower- and middle-income settings, where urban expansion, inadequate investment in air quality management, and insufficient enforcement of standards continue to impede meaningful improvement. In many such settings, ambient PM₂.₅ levels frequently exceed World Health Organization (WHO) recommended thresholds. Primary contributors include industrial emissions, vehicular exhaust, agricultural burning, and the widespread use of solid fuels for household energy needs, particularly in urbanized areas with pronounced socioeconomic disparities.

With advancing age, diminished physiological resilience and a higher burden of chronic disease render older adults especially susceptible to particulate matter exposure. Sustained PM₂.₅ exposure can elicit chronic, low-grade inflammation that aggravates cardiovascular stress. The presence of concurrent metabolic disorders—including hypertension, dyslipidemia, and diabetes—further magnifies these adverse effects, hastening cardiovascular disease development. Mechanistic studies also show that PM₂.₅ drives oxidative stress and endothelial dysfunction, triggers apoptosis of vascular cells, and facilitates thrombotic processes by stimulating platelet activation and impairing fibrinolysis (16, 17). Involvement of central mechanisms has been reported as well: sympathetic nervous system activation and disruption of the hypothalamic–pituitary–adrenal axis alter neuroendocrine and cardiovascular homeostasis (18). Hypoxia-induced pathways may additionally promote hypertension and arrhythmogenesis (19).

In addition to its well-recognized cardiovascular impact, long-term PM₂.₅ exposure has been implicated in a range of aging-related conditions that intersect biologically with ischemic heart disease. Inflammatory and oxidative responses induced by PM_2.5_ impair insulin sensitivity and promote insulin resistance, thereby elevating the risk of type 2 diabetes—a process that accelerates atherogenesis and worsens cardiac outcomes (5, 18). Evidence also links particulate pollution with chronic kidney disease, mediated by microvascular injury, renal inflammation, and endothelial dysfunction; as CKD is a powerful amplifier of cardiovascular risk, this association compounds the IHD burden (20, 21). Within the central nervous system, PM_2.5_ may traverse the blood–brain barrier and initiate neuroinflammation, microglial activation, and β-amyloid accumulation, pathological changes connected to dementia and cognitive decline (22, 23). Cognitive decline not only impairs daily functioning but also increases cardiovascular risk by reducing adherence to medical management. In addition, long-term exposure to PM₂.₅ has been linked to frailty in older adults, a condition likely driven by cumulative oxidative stress, chronic inflammation, and reduced physiological reserve. Frailty in turn predisposes individuals to cardiovascular complications and elevates the likelihood of adverse outcomes across multiple organ systems (24). In sum, research indicates that air pollution not only promotes the development of multiple chronic conditions in older adults but also adds to the overall cardiovascular disease burden.

We found that ischemic heart disease (IHD) from particulate matter exposure has generally declined in older adults (15). This improvement is likely tied to progress in healthcare, including better screening, quicker treatment, and stronger long-term control of chronic diseases such as hypertension, diabetes, lipid disorders, and IHD. As a result, many elderly people now live longer and enjoy better health even with multiple conditions. The decline is evident in lower disability-adjusted life years (DALYs) and more negative estimated annual percentage change (EAPC) values (24). Still, people aged 80 and older, though a smaller group, continue to show much higher baseline levels of disease burden than younger adults. Even relatively small improvements in healthcare—such as better drug management or more frequent check-ups—can bring large health benefits (25). In response to rapid population aging, many countries have directed more policy attention and resources toward older adults. Expanded health insurance, wider availability of community-based care, and rehabilitation services have all helped improve health outcomes and extend years of healthy life (26). When looking at sex differences, men consistently bear a heavier IHD burden related to particulate matter, particularly PM₂.₅, with higher rates of death and DALYs than women. This difference likely reflects the combined effects of biology, lifestyle, and environmental exposure. Biologically, males typically have larger lung volumes and higher minute ventilation, leading to greater particulate inhalation under the same exposure levels (27). Some evidence suggests that females may have more reactive airways, but males are more prone to deep lung particle deposition due to anatomical differences (28). Occupationally, men are more likely to work in high-exposure industries like construction, transport, manufacturing, and mining that have high exposure to particulate matter. Additionally, middle-aged men and men generally have a greater prevalence of cardiovascular and metabolic diseases such as hypertension, dyslipidemia, and diabetes than women. These comorbidities enhance susceptibility to environmental stressors, and long-term PM2. 5 exposure can intensify the development of these diseases, leading to increased mortality and DALYs in males (29).

Global trend analysis shows that the burden of ischemic heart disease (IHD) attributable to ambient particulate matter is closely related to socioeconomic development levels (8). In this study, the Sociodemographic Index (SDI) was used as a proxy for broader developmental progress. Drawing on three decades of mortality and DALY data disaggregated by country and region, we identified a significant inverse correlation between SDI and IHD burden (*P* < 0.05), reinforcing previous findings that populations in lower-income settings bear a disproportionate share of pollution-related cardiovascular risk (30). Several systemic factors may underlie this disparity, including outdated energy infrastructure, weak pollution control frameworks, and limited access to preventive health services. In many countries with low SDI scores, household energy needs are still met through traditional biomass. For example, in sub-Saharan Africa, more than 890 million people continue to rely on firewood, crop residues, and animal dung for daily cooking. This practice contributes to widespread and chronic indoor air pollution caused by particulate matter exposure (31). Meanwhile, ongoing urbanization and industrialization have intensified outdoor air pollution. In these regions, emissions from industrial facilities, vehicle exhaust, and coal-fired power plants have emerged as major contributors to ambient particulate matter pollution, leading to persistently high exposure levels.

In comparison, those countries with advanced levels of socioeconomic development have achieved significant reductions in air pollution. Adoption of clean energy programs, application of strict motor vehicle emission standards, and intensification of industrial emission regulations have all contributed to declining ambient PM2. 5 levels. These changes have consequently resulted in a significant reduction in particulate matter exposure-associated ischemic heart disease (IHD) burdens (32). More detailed examination of population attributable fraction (PAF) of IHD from particulate matter pollution indicated different patterns of exposure between different regions with different levels of development. In low-SDI countries, use of solid fuels in households is still the major source of pollution, but in high-SDI regions, industrial emissions and automotive emissions are major contributors of ambient particulate matter pollution. Additionally, there are usually severe limitations in cardiovascular disease screening, early detection, and treatment in low-income countries. Basic IHD-related primary prevention practices like preventive health care services and chronic disease management programs are also not adequately covered. Systematic issues could further exacerbate health effects from ambient particulate matter pollution in these environments (29).

In conclusion, when considering individual sex-based predictions of ischemic heart disease (IHD) burden in the subsequent three decades (2022–2040), even though there is an overall projection of a declining trend, there is a suggestion of a temporary increase in burden attributed to ambient particulate matter pollution some time around 2035 (15). Given this possible future scenario, sustained interest remains in the public health effects of ambient particulate matter pollution. It is important to continue regularly reviewing and optimizing intervention strategies to effectively prevent a reversal of the declining trend and to address any possible upturn in IHD in future years (33).

This study constitutes the first attempt to undertake a meticulous spatiotemporal assessment of ischemic heart disease (IHD) caused by ambient particulate matter pollution in aging communities. It forms an important basis for evidence-based targeted public health policy in aging societies. Nevertheless, certain limitations must be considered. First, there remains a lack of data availability in poor and less-developed countries. Some areas, therefore, did not have enough primary data, so modeled estimations had to be used to fill these gaps, which may have increased the range of uncertainty. Second, IHD in the GBD 2021 dataset may not have strict laboratory confirmation, which could result in incorrect misclassification or underreporting of disease. Third, ambient particulate matter pollution characterization in GBD 2021 is fairly simplified and does not reflect all heterogeneity of particulate constituents, e. g., chemical constituents or ultrafine particles, that may have an effect on health. Finally, health effects of ambient particulate matter pollution stem from an interplay of various factors such as exposure intensity, individual susceptibility, behavioral factors, socioeconomic status, and policy settings that were not all explored here (34). Additional research should continue to explore these multidimensional interactions in an attempt to advance knowledge of ambient particulate matter pollution's contribution to IHD burden among vulnerable communities.

## 5 Conclusion

Between 1990 and 2021, global trends show a decline in ischemic heart disease (IHD) burden associated with ambient particulate matter (PM) pollution among elderly populations. However, significant disparities persist, with lower-income regions, particularly in the Middle East and Africa, continuing to experience a high—and in some cases increasing—burden. Moreover, elderly males consistently exhibit a greater vulnerability to PM-related cardiovascular outcomes. These findings underscore the urgent need for targeted, equity-driven public health interventions aimed at strengthening the resilience of high-risk groups. Sustained efforts to reduce PM exposure are essential to mitigate the long-term cardiovascular risks faced by aging populations worldwide.

## Data Availability

The original contributions presented in the study are included in the article/Supplementary Material, further inquiries can be directed to the corresponding authors.
